# The association of trastuzumab with atrial fibrillation and heart failure in breast cancer patients in routine clinical practice: a population-based propensity score matching and competing risk model analysis

**DOI:** 10.1007/s10549-022-06753-7

**Published:** 2022-12-31

**Authors:** Wen-Chi Wu, Chi-Cheng Huang, Yi-Fang Tsai, Yen-Shu Lin, Chin-Jung Feng, Yen-Jen Chen, Jiun-I. Lai, Ta-Chung Chao, Chun-Yu Liu, Ling-Ming Tseng

**Affiliations:** 1 Division of Medical Oncology, Department of Oncology, Taipei Veterans General Hospital, Taipei, Taiwan; 2 Division of Cell and Immunotherapy, Department of Oncology, Taipei Veterans General Hospital, Taipei, Taiwan; 3 Division of Hematology, Department of Medicine, Taipei Veterans General Hospital, Taipei, Taiwan; 4 Comprehensive Breast Center, Department of Surgery, Taipei Veterans General Hospital, Taipei, Taiwan; 5 Division of General Surgery, Department of Surgery, Taipei Veterans General Hospital, No. 201, Sec 2, Shih-Pai Road, Taipei, 112 Taiwan; 6 Institute of Epidemiology and Preventive Medicine, College of Public Health, National Taiwan University, Taipei, Taiwan; 7 School of Medicine, National Yang Ming Chiao Tung University, Taipei, Taiwan; 8 Institute of Clinical Medicine, School of Medicine, National Yang Ming Chiao Tung University, Taipei, Taiwan; 9 Division of Transfusion Medicine, Department of Medicine, Taipei Veterans General Hospital, No. 201, Sec 2, Shih-Pai Road, Taipei, 112 Taiwan

**Keywords:** Trastuzumab, Human epidermal growth factor receptor 2(HER2), Heart failure, Atrial fibrillation, Cardiomyopathies

## Abstract

**Purpose:**

Trastuzumab, a potent anti-human epidermal growth factor receptor 2 (HER2) monoclonal antibody, is conditionally reimbursed by the Taiwan National Health Insurance (NHI) for HER2-positive breast cancer (BC). Trastuzumab-induced cardiotoxicity studies have well characterized heart failure (HF) but fewer addressed arrhythmia, particularly the association of potential life threatening atrial fibrillation (Af) is poorly characterized. We aimed to study the trastuzumab-related risk of Af and HF using the claimed data of Taiwan NHI.

**Methods:**

A nationwide retrospective cohort of patients with BC from the Taiwan NHI reimbursement database from January 2007 to December 2016 was analyzed. Propensity score matching and competing risk model analysis were used for adjusting confounding concurrent medication or comorbidities and competing events. The HF study was used to validate the method used.

**Results:**

For Af, 12,472 trastuzumab users were matched with 12,472 non-trastuzumab users. For HF, 12,241 trastuzumab users and 12,241 non-users were enrolled. We found that trastuzumab users had significantly worse HF-free survival but not Af-free survival than non-trastuzumab users. In the competing risk analysis, the use of trastuzumab did not increase the risk of Af (hazard ratio [HR] 0.76, *P* = 0.0006) but was associated with HF (HR 1.19, *P* = 0.0052). The risk trends among stratifications by comorbidities and concurrent medication remained in similar directions for both Af and HF.

**Conclusion:**

Trastuzumab in real-world practice was associated with an increased risk of HF, but was not associated with an increased risk of Af in BC patients. Trastuzumab-induced arrhythmogenic effects may be masked by concurrent heart-protecting measures, more prominent roles of comorbidities or concurrent medications under real-world settings. Further studies are required.

**Supplementary Information:**

The online version contains supplementary material available at 10.1007/s10549-022-06753-7.

## Introduction

Amplification and overexpression of the human epidermal growth factor receptor 2 (HER2) drive the proliferation, aggression, and metastatic behaviors of the breast epithelial cells [1, 2]. Twenty to 25% of patients with invasive breast cancer (BC) have high HER2 expression [2, 3]. Trastuzumab is a recombinant humanized monoclonal antibody that binds to HER2, and has been approved in treating HER2-positive BCs [4–6]. In Taiwan, HER2 positive BC affected 1 in 120 women [7]. The Taiwan’s National Health Insurance (NHI) has reimbursed trastuzumab for HER2 positive BCs under several conditions: (1) One-year adjuvant and or neoadjuvant therapy for HER2-positive nodal positive early BC. (2) HER2-positive metastatic BC.

Cardiac toxicities had long been a concern when using HER2 receptor antagonists, especially trastuzumab. As early in 2002, after analyzing seven phase 2 or 3 studies regarding combination use of trastuzumab and other chemo-regimen in advanced disease, Seidman et al*.* suggested that trastuzumab was associated with congestive heart failure (CHF) [8]. Large randomized trials demonstrated that trastuzumab, combined with chemotherapy regimens, especially anthracycline-base, caused higher cardiac dysfunction and subclinical loss of left ventricular ejection fraction (LVEF) in patients with early BC [4, 9, 10]. According to the US Food and Drug Administration (FDA) package insert, trastuzumab might cause “left ventricular dysfunction, arrhythmias, hypertension (HTN), disabling cardiac failure, cardiomyopathy, and cardiac death.” [11].

Besides CHF, other cardiac insults were less mentioned until recently. Also, most of the trastuzumab-induced cardiotoxicity research has been focused on heart dysfunction rather than the detailed description or rhythm of arrhythmias [12]. Furthermore, the established data on trastuzumab on cardiotoxicity in Asia was still limited.

We evaluated the characteristics, risk factors, and prognosis of heart failure (HF) and atrial fibrillation (Af), the most common arrhythmias predisposed to severe thromboembolism effects, from the Taiwanese National Health Insurance Research Database (NHIRD).

## Methods

This study has been approved by the institutional review board of Taipei Veterans General Hospital and conducted following the Declaration of Helsinki. We implemented a nationwide retrospective cohort study extracting data from the patients having visits or hospitalizations for BC from Taiwan's NHIRD. The NHIRD consists of the medical fee claims; it was contributed by enrollees' medical utilization, including more than 99% of the entire population in Taiwan. All data were extracted via the Applied Health Research Data Integration Service from Taiwan National Health Insurance Administration (NHIA). All applications are reviewed for approval of data release. Data analysis was guided by the monitoring regulation guidelines of the Scientific Data Center of the Ministry of Health and Welfare, Taiwan. The application for data and processing of the data were all conducted via the service and application process were detailed described previously [13, 14].

Female patients who were visited or admitted with a diagnosis of BC for the first time between 2007 and 2016 were identified. The patients were excluded if the incidence of HF and Af preceding index date. Meanwhile, the BC diagnosis was confirmed by the catastrophic illness database, another part of NHIRD. A tissue proof with an immunohistochemistry staining for the HER2 protein of 3 + or positive on fluorescence in situ hybridization was required to confirm HER2 positive. Due to the regulation of HER2 reimbursement, this medication was reimbursed for satisfying the previous requirement. The patients were divided into two groups based on their use of trastuzumab, identified with specific drug codes in NHIRD, treated group, and untreated group, respectively. Af and HF were the primary outcomes in this study, and the follow-up for both groups began on the index date, and they followed up until the date of Af/HF hospitalization, death, or end of 2016. Because the chance of primary outcomes can be confounded by competing risks of mortality, a proportional hazards model for the cumulative incidence of a failure cause of primary outcomes, proposed by Fine and Gray [15], was conducted to evaluate the related hazards. Propensity scores, the probability of evaluating a patient to the treated group given control covariates, were implemented using greedy matching. The control covariates included age, comorbidities, and medications.

Demographic data included age, comorbidities and associated medications. The comorbidities listed by ICD 9 or ICD 10 codes (Supplement 1) included HTN, diabetes mellitus (DM), chronic kidney diseases (CKD), and hyperlipidemia or dyslipidemia. The medication used was defined by the Anatomical Therapeutic Chemical Classification system proposed World Health Organization, including aspirin, beta-blockers, renin-angiotensin system (angiotensin-converting enzyme inhibitor, ACEI/angiotensin receptor blocker, ARB), non-steroid anti-inflammatory drugs (NSAIDs), statins, and metformin. The codes for the medication mentioned above were demonstrated in Supplement 2. Medication use was defined as the cumulated prescription days of more than 90 days.

To clarify the association between the cardiotoxic anthracyclines and cardiac outcomes, we further analyzed the frequency of cardiac examinations and the relationship between BC-specific anthracyclines and Af/HF in enrolled patients. The examinations for HF included echocardiography with or without color Doppler, ventricular ejection fraction (EF) or multi-gated acquisition (MUGA) radionucleotide scanning. The examinations to detect Af included 12-lead-electrocardiogram (ECG), Treadmill-exercise ECG, and 24 h Holter ECG monitoring.

Data management and analysis were performed using SAS 9.4 software (SAS Institute Inc). Calculated results were presented as the estimated number and the 95% confidence.

## Results

A total of 100,861 women diagnosed with BC without known Af and 99,283 women without HF before the index date from 2007–2016 were enrolled for propensity score matching. Baseline demographic characteristics of patients before and after the matching were illustrated in Table 1. There were a substantial proportion of BC patients with comorbidities in the entire cohort, including 19.7% patients with DM, 17.1% patients with hyperlipidemia, up to 33.9% patients with HTN, and a much smaller proportion with CKD (Table 1a and b).

**Table 1:** Comorbidities and concurrent medication use of breast cancer patients with and without trastuzumab, before and after matching (a) Heart failure (b) Atrial fibrillation

A	Before matching							After matching					
		Non-Trastuzumab		Trastuzumab		Chi-sqaure	*p*-value	Non-Trastuzumab		Trastuzumab		Chi-square	*p*-value
		No	%	No	%			No	%	No	%		
	Total	87,041	100	12,242	100			12,241	100	12,241	100		
DM	Yes	16,801	19.3	2247	18.4	6.22	0.013	2124	17.4	2247	18.4	4.21	0.040
	No	70,240	80.7	9995	81.6			10,117	82.6	9994	81.6		
Hyperlipidemia	Yes	14,904	17.1	1814	14.8	40.72	< 0.0001	1815	14.8	1814	14.8	0.00	0.986
	No	72,137	82.9	10,428	85.2			10,426	85.2	10,427	85.2		
Hypertension	Yes	28,953	33.3	3,898	31.8	9.81	0.002	3,693	30.2	3,898	31.8	8.02	0.005
	No	58,088	66.7	8344	68.2			8548	69.8	8343	68.2		
CKD	Yes	2607	3.0	297	2.4	12.24	0.001	285	2.3	297	2.4	0.25	0.615
	No	84,434	97.0	11,945	97.6			11,956	97.7	11,944	97.6		
Aspirin	Yes	8336	9.6	963	7.9	37.00	< 0.0001	934	7.6	963	7.9	0.48	0.488
	No	78,705	90.4	11,279	92.1			11,307	92.4	11,278	92.1		
NSAID	Yes	45,224	52.0	6180	50.5	9.35	0.002	6078	49.7	6180	50.5	1.70	0.192
	No	41,817	48.0	6062	49.5			6163	50.3	6061	49.5		
Statins	Yes	17,254	19.8	2170	17.7	29.99	< 0.0001	2105	17.2	2170	17.7	1.20	0.274
	No	69,787	80.2	10,072	82.3			10,136	82.8	10,071	82.3		
Metformin	Yes	11,066	12.7	1522	12.4	0.77	0.382	1447	11.8	1,521	12.4	2.10	0.147
	No	75,975	87.3	10,720	87.6			10,794	88.2	10,720	87.6		
Beta-blockers	Yes	17,788	20.4	2380	19.4	6.57	0.010	2266	18.5	2380	19.4	3.45	0.063
	No	69,253	79.6	9,862	80.6			9975	81.5	9861	80.6		
ACEI/ARBs	Yes	17,578	20.2	2290	18.7	14.87	0.000	2211	18.1	2290	18.7	1.70	0.192
	No	69,463	79.8	9952	81.3			10,030	81.9	9951	81.3		
Age	Mean(y)	53.06		52.50		5.51	< 0.0001	52.42		52.50		0.61	0.543
	STD	10.65		10.29				10.49		10.28			

B	Before matching							After matching					
		Non-Trastuzumab		Trastuzumab		Chi-square	*p*-value	Non-Trastuzumab		Trastuzumab		Chi-sqaure	*p*-value
		No	%	No	%			No	%	No	%		
	Total	88,388	100	12,473	100			12,472	100	12,472	100		
DM	Yes	17,532	19.3	2,350	18.8	6.83	0.009	2309	18.5	2350	18.8	0.44	0.505
	No	70,856	80.2	10,123	81.2			10,163	81.5	10,122	81.2		
Hyperlipidemia	Yes	15,399	17.4	3,876	15.0	43.68	< 0.0001	1875	15.0	1,876	15.0	0.00	0.986
	No	72,989	82.6	10,597	85.0			10,597	85.0	10,596	85.0		
Hypertension	Yes	30,091	34.0	4066	32.6	10.20	0.001	3861	31.0	4,066	32.6	7.77	0.005
	No	58,297	66.0	8,407	67.4			8611	69.0	8406	67.4		
CKD	Yes	2942	3.3	336	2.7	14.00	0.000	316	2.5	336	2.7	0.63	0.427
	No	85,446	96.7	12,137	97.3			12,156	97.5	12,136	97.3		
Aspirin	Yes	9012	10.2	1057	8.5	36.05	< 0.0001	1048	8.4	1057	8.5	0.04	0.838
	No	79,376	89.8	11,416	91.5			11,424	81.6	11,415	91.5		
NSAID	Yes	46,234	52.3	6358	51.0	7.79	0.005	6246	50.1	6358	51.0	2.01	0.156
	No	42,154	47.7	6115	49.0			6226	49.9	6114	49.0		
Statins	Yes	17,962	20.3	2275	18.2	29.55	< 0.0001	2,237	17.9	2275	18.2	0.39	0.532
	No	70,426	79.7	10,877	81.8			10,235	82.1	10,197	81.8		
Metformin	Yes	11,543	13.1	1596	12.8	0.67	0.413	1613	12.9	1595	12.8	0.12	0.734
	No	76,845	86.9	10,877	87.2			10,859	87.1	10,877	87.2		
Beta-blockers	Yes	18,691	21.1	2534	20.3	4.54	0.033	2471	19.8	2534	20.3	0.99	0.319
	No	69,697	78.9	9939	79.7			10,001	80.2	9938	79.7		
ACEI/ARBs	Yes	18,553	21.0	2440	19.6	13.53	0.000	2397	19.2	2,440	19.6	0.47	0.491
	No	69,835	79.0	10,033	80.4			10,075	80.8	10,032	80.4		
Age	Mean(y)	53.24		52.65		5.71	< 0.0001	52.71		52.65		0.39	0.696
	STD	10.73		10.35				10.58		10.35			

*DM* diabetes mellitus, *CKD* chronic kidney disease, *NSAID* non-steroid anti-inflammatory drug, *ACEI* angiotensin converting enzyme inhibitor, *ARB* angiotensin receptor blockers

**Fig. 1:**
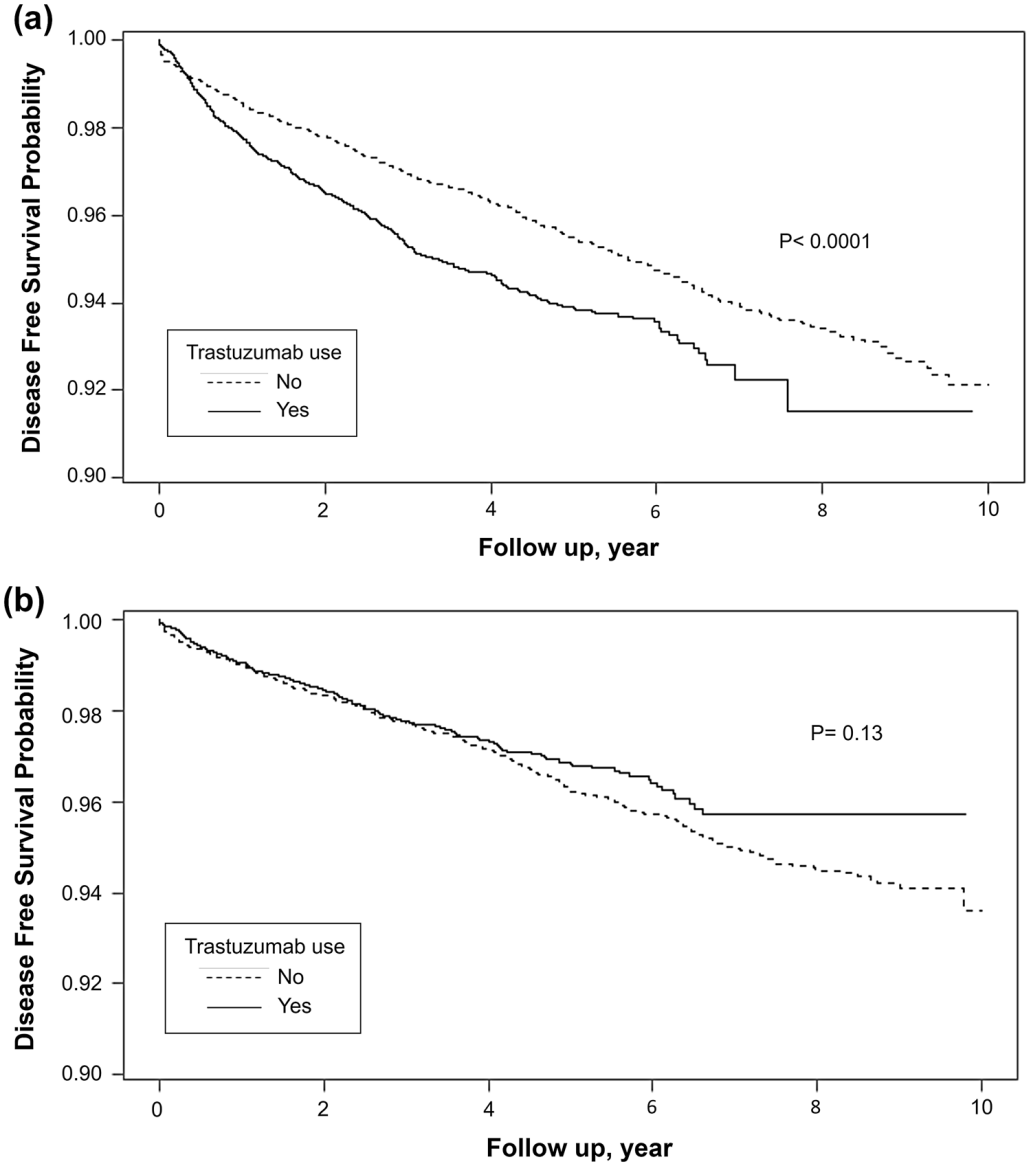
Months from Herceptin start to heart failure (**a**) and atrial fibrillation (**b**)

Kaplan–Meier curves demonstrating HF-free and Af-free survival in patients with and without trastuzumab are illustrated in Fig. 1a and b, respectively. After matching, patients who received trastuzumab treatment had worse HF-free survival than non-trastuzumab patients (*P* < 0.0001). In contrast, a patient who received trastuzumab treatment was not associated with inferior Af-free survival (*P* = 0.13).

**Fig. 2:**
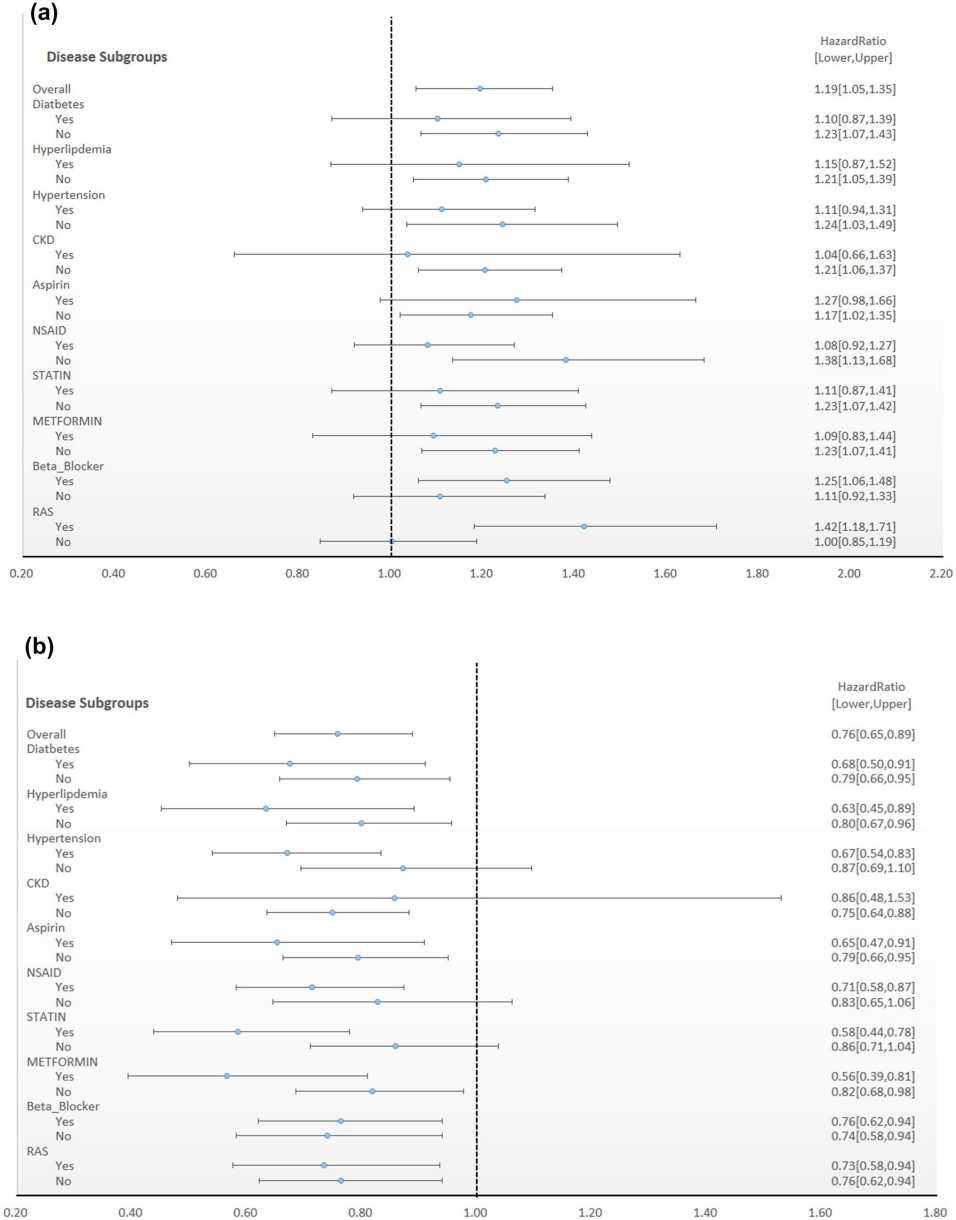
Multivariate stratified analysis of the association between trastuzumab use and heart failure (**a**) and atrial fibrillation (**b**). The hazard ratio (HR) and 95% confidence interval (CI) of the difference in risk of getting risks between trastuzumab and non-trastuzumab groups were shown

Next, we performed a competing risk analysis to estimate the effects of trastuzumab on HF and Af by considering death as a competing event to HF or Af after adjusting for comorbidities and concurrent medications. Tables 2a and b estimate the effect of trastuzumab on HF and Af from multivariate cox proportional hazards models. As shown in Table 2, after adjusting for age, DM, HTN, hyperlipidemia, CKD, Aspirin, NSAIDs, Statins, metformin, beta-blockers, and renin-angiotensin system (angiotensin-converting enzyme inhibitor, ACEI/angiotensin receptor blocker, ARB) drugs, trastuzumab treatment is associated with increased risk of HF, with an estimated hazard ratio (HR) of 1.193 (95% confidence interval [CI] 1.054–1.351, *P* = 0.0052). In contrast, trastuzumab treatment is not associated with an increased risk of Af (HR = 0.759 [95 CI 0.649–0.889], *P* = 0.0006). We also examined the risk of trastuzumab in each subgroup stratifications stratified by comorbidities and concurrent medications (Fig. 2a and b). As shown in Fig. 2a, the increased risk of HF by trastuzumab treatment remained in similar trends in each subgroups stratified by comorbidities and concurrent medication. In contrast, Fig. 2b demonstrates a decreased risk of Af by trastuzumab with a similar trend in each stratified subgroups. Interestingly, wider ranges of confidence interval of hazards were seen in patients with CKD, in both analyses of Af and HF, probably due to much smaller numbers of patients with CKD.

**Table 2:** Multivariate cox proportional hazards with competing risk analysis models for breast cancer patients developing (a) heart failure (b) atrial fibrillation

(a)	Heart failure			
	HR	95% C.I		*p*-value
		L	U	
Trastuzumab	1.193	1.054	1.351	0.005
Age	1.012	1.004	1.019	0.002
DM	0.951	0.750	1.205	0.6769
HTN	0.831	0.690	1.000	0.0494
Hyperlipidemia	0.724	0.558	0.938	0.0146
CKD	1.673	1.306	2.144	< 0.0001
Aspirin	1.451	1.214	1.734	< 0.0001
NSAID	0.990	0.863	1.134	0.8815
Statin	0.776	0.642	0.939	0.0091
Metformin	1.022	0.790	1.323	0.8666
Beta-blockers	4.087	3.401	4.911	< 0.0001
ACEI/ARB	2.078	1.631	2.647	< 0.0001

(b)	Atrial fibrillation			
	HR	95% C.I		*p*-value
		L	U	
Trastuzumab	0.758	0.648	0.888	0.0006
Age	1.009	1.001	1.018	0.0359
DM	0.808	0.594	1.101	0.1771
HTN	0.895	0.718	1.114	0.3198
Hyperlipidemia	0.752	0.578	0.978	0.0334
CKD	1.500	1.108	2.032	0.0088
Aspirin	1.602	1.283	2.000	< 0.0001
NSAID	0.991	0.836	1.174	0.9147
Statin	1.129	0.899	1.418	0.2964
Metformin	1.061	0.760	1.481	0.7294
Beta-blockers	4.299	3.435	5.381	< 0.0001
ACEI/ARB	1.396	1.088	1.792	0.0088

*C.I* confidential interval, *HR* hazard ratio, *DM* diabetes mellitus, *HTN* hypertension, *CKD* chronic kidney disease, *NSAID* non-steroid anti-inflammatory drug, *ACEI* angiotensin converting enzyme inhibitor, *ARB* angiotensin receptor blockers

Further evaluations of the effects of BC-specific anthracyclines were shown in Supplement 3 and Supplement 4. In supplement 3, there were slightly more anthracyclines usage in non-trastuzumab patients, reflecting the real-world practice to avoid concomitant trastuzumab and anthracycline treatment. The proportion of cardiac examinations for cardiac ejection function and rhythms, doxorubicin (including conventional and liposomal doxorubicin), and epirubicin were similar in Af and HF groups (Supplement 3). After adjusted for comorbidities, concurrent medications including anthracyclines (doxorubicin and epirubicin), trastuzumab treatment is still significantly associated with increased risk of HF, but is not associated with an increased risk of Af, the hazard ratios of trastuzumab treatment on outcome were similar compared to Table 2a and b (Table 2a and b, and Supplement 4).

## Discussions

Cardiotoxicities induced by trastuzumab have long been established. In this study, we utilized the Taiwan NHIRD data to evaluate the risk of Af and HF in patients treated with trastuzumab vs. matched-non-trastuzumab controls. We found trastuzumab users were associated with worse HF-free survival and increased association with HF in a competing risk analysis, but this was not seen with Af.

Considering the impacts on cardiac outcomes on concurrent cardiovascular agents, we analyzed how aspirin, NSAIDs, statins, metformin, beta-blockers, and RAAS inhibitors affect the heart AEs. Aspirin, beta-blockers, and RAAS inhibitors were associated with a higher incidence of heart failure and atrial fibrillations. It was impressive because beta-blockers and RAAS agents were standard treatments for CHF. One possible reason was that patients taking those drugs were prone to have AEs. Aspirin, NSAIDs, statins, metformin, beta-blockers, and RAAS drugs might represent the severity of the underlying diseases. In the subgroup analysis, we found that metformin and statins therapy was associated with a lower incidence of Af. The results were partially consistent with previous studies that statin therapy [16] and metformin [17] were significantly associated with a reduced risk of incidence or recurrence of Af. Aspirin was not recommended to prevent stroke in patients with Af as monotherapy [18] and might result in the association of aspirin and lower Af rate.

Unexpectedly, the results revealed that the trastuzumab group had a lower risk of developing Af than the control arm. We believed the result was valid since a similar population was analyzed for HF. The result was consistent with the previous studies that trastuzumab was associated with increased heart failure incidence. Prior Taiwan NHIRD-based studies have also examined the potential confounding effects of anthracyclines on the cardiotoxicities [19, 20]. Chang et al. [19] found BC patients receiving trastuzumab had an increased risk of major adverse cardiac and cerebrovascular event (MACCE), especially HF, a similar but some different outcome endpoint compared to our current study. Chang et al. revealed trastuzumab-induced risk of MACCE is independent of anthracyclines. Similarly, Chieh et al. [20] studied an earlier NHIRD cohort and disclosed that both anthracyclines and trastuzumab contribute to increased risk of HF and cardiomyopathy. Accordingly, our additional analysis (Supplement 3 and 4) revealed that there were slightly more anthracyclines usage in non-trastuzumab patients, reflecting the real-world practice to avoid concomitant trastuzumab and anthracycline treatment. After being adjusted for comorbidities, concurrent medication including anthracyclines, trastuzumab treatment is associated with increased risk of HF, but is not associated with an increased risk of Af. This additional analysis also supported prior NHIRD-based studies which showed trastuzumab-induced HF risk is independent to anthracyclines. Moreover, it is possible that transient arrhythmia during trastuzumab might have been missed and not coded in the NHIRD claims. This is apparently a limitation of this NHIRD-based study [13, 14]. Nevertheless, we additionally examined the correlated cardiac exams among trastuzumab and matching non-trastuzumab users (Supplement 3), we found similar frequency patterns of cardiac examinations among Af and HF cohorts. Under these similar frequency patterns of cardiac examinations, the trastuzumab-associated risks for HF and Af still showed a distinct difference in hazard ratios (Supplement 3 and 4).

Recently, a meta-analysis from 15 studies involving 8124 patients has reported an overall 1.2% incidence of Af in BC patients receiving trastuzumab. The incidence seemed not influenced by the formulation of trastuzumab, the additional use of neoplastic agents, anthracycline exposure status, or concurrent radiotherapy [21]. There have been several in vitro mechanistic studies showing arrhythmogenic effects of trastuzumab under various doses [22–25]. In contrast, a minority reported trastuzumab at a concentration of 10 mg/L did not trigger more arrhythmias in rat hearts [26]. Unspecified arrhythmias were reported in the relatively little literature regarding arrhythmias in trastuzumab use in HER2 high expression BC patients. As early as 2001, Behr et al. tried to approach trastuzumab-induced cardiotoxicity by applying 111In-DTPA-trastuzumab to 20 patients. Only one developed arrhythmia among the seven patients showing isotope uptake in myocardium on image [27]. In one small cohort including 27 metastatic BC patients, trastuzumab was believed to cause arrhythmias in 6 patients, one of which developed sinus bradycardia and others were unspecified [28]. In a larger study including 68,113 BC patients, the chemotherapy group was shown to have higher Af incidence, while in the subgroup analysis, there were no significant differences regarding trastuzumab use [29]. Other abnormal electrocardiogram findings, including branch bundle blocks, QT prolonged, ventricular bigeminy, and non-sustained ventricular tachycardia in HER2-expressed BC patients, were shown as case reports [30–32]. A small cohort of 20 metastatic HER2 BC patients reported no arrhythmias after trastuzumab infusion [33]. The evidence regarding arrhythmias associated with trastuzumab in BC patients was still vague, controversial, and not well-established. According to the above pre-clinical and clinical evidence, we suggested that the AE of arrhythmias of trastuzumab might refer to ventricular tachycardia and ventricular fibrillation rather than Af.

There were some limitations to our study. First, the misclassification of false-positive diagnosis of administrative codes for HF and Af might occur as limitations of population-based, including administrative data. Second, more frequent cardiac screening in patients on trastuzumab led to detection bias. Third, education in patients receiving trastuzumab might induce health behavior bias. No information regarding the value of LVEF and the severity of the cardiac outcomes were available using the NHIRD. Also, this study only detected patients who used NHI-supported trastuzumab; those who paid for the regimen themselves were omitted. EF is not a routine examination in real-world clinical practice in non-HER2 BC patients. Better comparisons across exposure groups could be achieved if the evaluation of heart function was performed in non-HER2 patients. Moreover, transient arrhythmia during trastuzumab infusion might have been missed and not coded in the NHIRD claims.

Furthermore, although we attempted to match both groups with age, comorbidities including DM, HTN, hyperlipidemia, CKD, medication usage including aspirin, NSAIDs, statins, metformin, beta-blockers, and drugs against RAAS system, residual confounding still existed. Our study reflects the real-world clinical practice of trastuzumab treatment in Taiwan under the NHI coverage. Our data showed that around 12% of all BC patients had received trastuzumab. HER2-positivity accounts for 20–25% of all BC patients. Patients with HER2-positive node-negative early BC may have never received trastuzumab according to the reimbursement criteria. Nevertheless, these patients would still receive cardiotoxic chemotherapy (such as anthracyclines) and thus may have risks of Af. Moreover, other well-characterized risk factors for Af, such as HTN and DM, and concurrent medications may have masked the less prominent role of trastuzumab in developing Af.

Collectively, the opposite result of trastuzumab on the risk of Af despite its known arrhythmogenic effect may be explained by several reasons below: (1) In general, HF can increase the risk of developing Af subsequently due to structural atrial stretch and interstitial fibrosis [34]. In contrast, most trastuzumab-induced heart dysfunction can be improved or reversible [35, 36] and therefore Af would not precede HF. (2) In the real-world setting, clinicians would try to avoid cardiotoxic agents when prescribing trastuzumab, theoretically lowering the incidence of cardiotoxicity. (3) Taiwanese patients with HER2-positive node-negative early BC may have never received trastuzumab according to the reimbursement criteria. Nevertheless, these patients (non-trastuzumab users) would still receive cardiotoxic chemotherapy (such as anthracyclines) or radiotherapies and thus may have risks of Af. Moreover, other well-characterized risk factors for Af, such as HTN and DM, and concurrent medications may have masked the less prominent role of trastuzumab in developing Af.

Indeed, clinical reports have been focused on the more prominent cardiomyopathic role of trastuzumab; the incidence of Af (1.2%) is lower than that of HF (1–4%) [37, 38]. Nevertheless, given the possible arrhythmogenic effects of trastuzumab [12], further studies are necessary to characterize the potential electrophysiological side effects.

## Conclusions

Trastuzumab in real-world patients was associated with an increased risk of HF, but was not associated with an increased risk of Af in BC patients. Trastuzumab-induced arrhythmogenic effects may be masked by heart-protecting measures, more prominent roles of comorbidities, or concurrent medications under real-world settings. Further studies are required.

## Supplementary Information

Below is the link to the electronic supplementary material.

Supplementary file1 (DOCX 19 KB)

## Data Availability

The datasets supporting the conclusions of this article are available in the National Health Insurance Research Database, Taiwan (NHIRD) [https://nhird.nhri.org.tw/en/index.html]. The use of NHIRD is limited to research purposes only. Applicants must follow the related regulations of National Health Insurance Administration and NHRI (National Health Research Institutes). The datasets produced and analyzed during the present study are available from the corresponding authors upon reasonable request.
