# Supplementary material for: The association of trastuzumab with atrial fibrillation and heart failure in breast cancer patients in routine clinical practice: a population-based propensity score matching and competing risk model analysis
Source: Breast Cancer Res Treat. 2022 Dec 31;198(1):113–22. doi: 10.1007/s10549-022-06753-7 (PMC9883308; doi:10.1007/s10549-022-06753-7)
Supplement: Supplementary file 1 — Supplementary file1 (DOCX 19 KB) [file 10549_2022_6753_MOESM1_ESM.docx]

**Supplement 1. ICD codes**

Breast cancer ICD 9: 174, 175, ICD10: C50

Heart failure ICD 9: 428, ICD 10: I50

Atrial fibrillation ICD 9: 427.32, ICD 10: I480, I482, I489.1

Hypertension ICD 9: 40-405, ICD 10: I10-13, I15

Diabetes mellitus ICD 9: 250, ICD 10: E08-E13

Chronic kidney disease ICD 9: 585, ICD 10: N18

Hyperlipidemia or dyslipidemia ICD 9: 272.0-272.2, ICD 10: E780-782

**Supplement 2. Anatomical Therapeutic Chemical Classification system proposed World Health Organization**

Aspirin: B01AC06, B01AC56

Non-steroid anti-inflammatory drug (NSAID): M01A, M01B, NOBA, C01EB16

Statins: C10AA, C10BX, C10BA, A10BH51

Metformin: A10BA02, A10BD13, A10BD11, A10BD10, A10BD08, A10BD05, A10BD03

Beta-blockers: C07

Angiotensin-converting enzyme (ACE) inhibitors: C09

Doxorubicin (including conventional doxorubicin and liposomal doxorubicin): L01DB01

Epirubicin: L01DB03

Trastuzumab: KC009612B5, KC006252B5, K0006252B5, K0009612B5, K000790261

**Supplement 3. Comorbidities and concurrent medication use in breast cancer patients with and without trastuzumab (matching cohort)**

|  | Trastuzumab users | | Non-trastuzumab users | |
| --- | --- | --- | --- | --- |
|  | No. | % | No. | % |
| Heart Failure | 12,241 |  | 12,247 |  |
| Doxorubicin* | 1,872 | 15.3 | 2,037 | 16.6 |
| Epirubicin | 3,231 | 26.4 | 4,525 | 36.9 |
| Exams for ejection fraction | 625 | 5.1 | 440 | 3.6 |
| Exams for rhythm | 7,440 | 60.8 | 8,366 | 68.3 |
| Atrial fibrillation | 12,472 |  | 12,472 |  |
| Doxorubicin* | 1,909 | 15.3 | 2,005 | 16.1 |
| Epirubicin | 3,282 | 26.3 | 4,500 | 36.1 |
| Exams for ejection fraction | 644 | 5.2 | 419 | 3.4 |
| Exams for rhythm | 7,608 | 61.0 | 8,608 | 69.0 |

*Doxorubicin: includes conventional and liposomal doxorubicin

**Supplement 4. Multivariate cox proportional hazards with competing risk analysis models regarding trastuzumab with additional adjusting anthracyclines for breast cancer patients developing (a) heart failure (b) atrial fibrillation**

| (a) | **Heart failure** | | | |
| --- | --- | --- | --- | --- |
|  | | 95% C.I. | |  |
|  | HR | L | U | p-value |
| Trastuzumab | 1.214 | 1.072 | 1.375 | 0.002 |
| Age | 1.013 | 1.005 | 1.020 | 0.001 |
| DM | 0.949 | 0.748 | 1.203 | 0.665 |
| HTN | 0.721 | 0.557 | 0.934 | 0.013 |
| Hyperlipidemia | 0.831 | 0.690 | 1.000 | 0.050 |
| CKD | 1.684 | 1.314 | 2.157 | <0.0001 |
| Aspirin | 1.456 | 1.219 | 1.740 | <0.0001 |
| NSAID | 0.989 | 0.863 | 1.134 | 0.878 |
| Statin | 0.780 | 0.644 | 0.943 | 0.010 |
| Metformin | 1.020 | 0.788 | 1.320 | 0.879 |
| Beta-blocker | 4.094 | 3.409 | 4.917 | <0.0001 |
| ACEI/ARB | 2.080 | 1.634 | 2.649 | <0.0001 |
| Doxorubicin* | 1.053 | 0.889 | 1.247 | 0.553 |
| Epirubicin | 1.181 | 1.035 | 1.348 | 0.014 |

*Doxorubicin: included conventional and liposomal doxorubicin

| (b) | **Atrial fibrillation** | | | |
| --- | --- | --- | --- | --- |
|  | | 95% C.I. | |  |
|  | HR | L | U | p-value |
| Trastuzumab | 0.767 | 0.655 | 0.899 | 0.001 |
| Age | 1.010 | 1.001 | 1.019 | 0.025 |
| DM | 0.808 | 0.593 | 1.100 | 0.176 |
| HTN | 0.750 | 0.577 | 0.975 | 0.032 |
| Hyperlipidemia | 0.894 | 0.717 | 1.113 | 0.315 |
| CKD | 1.510 | 1.115 | 2.045 | 0.008 |
| Aspirin | 1.608 | 1.287 | 2.008 | <0.0001 |
| NSAID | 0.990 | 0.836 | 1.174 | 0.911 |
| Statin | 1.129 | 0.899 | 1.418 | 0.298 |
| Metformin | 1.056 | 0.756 | 1.475 | 0.748 |
| Beta-blocker | 4.297 | 3.433 | 5.377 | <0.0001 |
| ACEI/ARB | 1.400 | 1.091 | 1.796 | 0.008 |
| Doxorubicin* | 1.029 | 0.833 | 1.271 | 0.790 |
| Epirubicin | 1.131 | 0.957 | 1.336 | 0.149 |

*Doxorubicin: included conventional and liposomal doxorubicin

C.I., confidential interval; HR, hazard ratio; DM, diabetes mellitus; HTN, hypertension; CKD, chronic kidney disease; NSAID, non-steroid anti-inflammatory drug; ACEI, angiotensin converting enzyme inhibitor; ARB, angiotensin receptor blockers
